# Improved Postoperative Outcomes after Prehabilitation for Colorectal Cancer Surgery in Older Patients: An Emulated Target Trial

**DOI:** 10.1245/s10434-022-12623-9

**Published:** 2022-10-05

**Authors:** Thea C. Heil, Emiel G. G. Verdaasdonk, Huub A. A. M. Maas, Barbara C. van Munster, Marcel G. M. Olde Rikkert, Johannes H. W. de Wilt, René J. F. Melis

**Affiliations:** 1 Department of Geriatric Medicine, Radboud University Medical Center, Nijmegen, The Netherlands; 2 Department of Surgery, Jeroen Bosch Hospital, ‘s-Hertogenbosch, The Netherlands; 3 Department of Geriatric Medicine, Elisabeth-Tweesteden Hospital, Tilburg, The Netherlands; 4 Department of Internal Medicine, University Medical Center Groningen, University of Groningen, Groningen, The Netherlands; 5 Department of Surgery, Radboud University Medical Center, Nijmegen, The Netherlands

## Abstract

**Background:**

The aim of this study was to assess the effect of a multimodal prehabilitation program on perioperative outcomes in colorectal cancer patients with a higher postoperative complication risk, using an emulated target trial (ETT) design.

**Patients and Methods:**

An ETT design including overlap weighting based on propensity score was performed. The study consisted of all patients with newly diagnosed colorectal cancer (2016–2021), in a large nonacademic training hospital, who were candidate to elective colorectal cancer surgery and had a higher risk for postoperative complications defined by: age ≥ 65 years and or American Society of Anesthesiologists score III/IV. Intention-to-treat (ITT) and per-protocol analyses were performed to evaluate the effect of prehabilitation compared with usual care on perioperative complications and length of stay (LOS).

**Results:**

Two hundred fifty-one patients were included: 128 in the usual care group and 123 patients in the prehabilitation group. In the ITT analysis, the number needed to treat to reduce one or more complications in one person was 4.2 (95% CI 2.6–10). Compared with patients in the usual care group, patients undergoing prehabilitation had a 55% lower comprehensive complication score (95% CI −71 to −32%). There was a 33% reduction (95% CI −44 to −18%) in LOS from 7 to 5 days.

**Conclusions:**

This study showed a clinically relevant reduction of complications and LOS after multimodal prehabilitation in patients undergoing colorectal cancer surgery with a higher postoperative complication risk. The study methodology used may serve as an example for further larger multicenter comparative effectiveness research on prehabilitation.

**Supplementary Information:**

The online version contains supplementary material available at 10.1245/s10434-022-12623-9.

The goal of prehabilitation of cancer patients indicated for surgery is to improve recovery after treatment to reduce the severity of treatment-related complications that may cause significant disability.^1^ Prehabilitation can be offered as a single modality intervention, consisting of only exercise training, or as a multimodal intervention including exercise training as well as nutrition assessment and reduction of intoxications (smoking and alcohol use) and psychological stress.^2^ Although mortality after colorectal cancer surgery has already decreased significantly in recent years, older patients and patients with a higher American Society of Anesthesiologists (ASA) score are still prone to postoperative complications.^3,4^ Therefore, these patients in particular could benefit from prehabilitation.

While the prehabilitation concept appears promising, there is conflicting scientific evidence for its effectiveness.^5–9^ Several RCTs have been performed, showing different effects of multimodal prehabilitation, ranging from meaningful changes in postoperative functional walking capacity and significantly improved postoperative clinical outcomes,^10,11^ to no effect on postoperative outcomes.^12^

Because these RCTs are usually performed under ideal circumstances, including only a selected group of patients, this can compromise external validity.^11–13^ Studies based on observational, real-world data could be of added value to better understand for whom prehabilitation may be of benefit. However, as a result of the absence of a strict research protocol and randomization, the outcomes of observational studies are more prone to bias.^14^

To minimize the impact of bias in observational studies for intended effects, Hernán and Robins described the emulated target trial (ETT) design, in which observational data are used to mimic an RCT as close as possible.^15^ The ETT enables a more accurate comparison of clinical trial and real-world data, because it applies the same eligibility criteria as the target trial (RCT) and reduces biases by a well-defined time zero.^15^ Moreover, ETTs can typically include a more heterogeneous group of patients.

The aim of this study was to evaluate the effect of a multimodal prehabilitation program compared with usual care on perioperative outcomes in patients undergoing elective colorectal surgery with a higher postoperative complication risk, using an ETT design.

## Patients and Methods

### Data Sources

Patients diagnosed with newly onset colorectal cancer, between January 2016 and July 2021, in a large nonacademic training hospital (the Jeroen Bosch Hospital, ‘s-Hertogenbosch, The Netherlands) were identified from the Netherlands Cancer Registry (NCR). Prehabilitation was not offered in this hospital before 1 June 2017. All patients with a multidisciplinary team (MDT) consultation before this time were therefore included in the control group. Before prehabilitation was offered to every patient meeting the inclusion criteria from November 2018, several pilot studies were conducted. To reduce bias, all patients with an MDT consultation between 1 June 2017 and 31 October 2018 were excluded. Furthermore, patients with an MDT consultation date between 16 March 2020 and 8 June 2020 or a part of the preoperative period (time between MDT consultation and surgery) within this period were also excluded because prehabilitation was not offered during this period as a result of COVID-19. Patients with MDT dates between 1 November 2018 and 15 March 2020, and 9 June 2020 and 31 July 2021 are therefore included in the intervention group.

On the basis of electronic patient records, information on comorbidities, polypharmacy, substance use, physical status, and adherence to the prehabilitation program was collected. Information on ethnicity was not available.

### Study Design

The ETT framework was used to design this single-center study.^15^ The target trial protocol, modified from two previous RCT protocols,^11,16^ and the emulation of this target trial is summarized in Supplement 1.

The medical ethics committee region Brabant (file number NW2021-02) advised that this study did not fall within the remit of the Medical Research Involving Human Subjects Act (WMO).

### Eligibility Criteria

The inclusion criteria defined in the target trial protocol could be applied to the ETT protocol (Supplement 1). Candidacy for elective surgery was determined based on the preoperative MDT consultation involving surgeons, oncologists, a radiologist, a radiotherapist, a pathologist, and a nurse practitioner discussing the need for surgery. The exclusion criteria were implemented with some deviations from the target protocol (Supplement 1) because the target trial using observational data was emulated. Because there was no measure to define patients with an unstable cardiac or respiratory disease, patients were excluded from the ETT protocol if doubts were described about the operability (on the basis of patients’ physical condition, not tumor characteristics) during MDT consultation. In the target trial protocol, patients with locomotor limitations precluding exercise training and patients with cognitive deterioration impeding adherence to the program were excluded. This was translated to the ETT protocol as exclusion of patients who were wheelchair dependent or who had dementia registered as a comorbidity, respectively. The ability to offer prehabilitation for a minimum of 4 weeks was translated into the ETT protocol as exclusion of patients for whom the first MDT consultation took place after surgery or for whom there was no MDT consultation at all. Patients were also excluded if tumor characteristics, such as an obstructive tumor, a bleeding carcinoma, a signet ring cell carcinoma, and/or severe tumor related pain, did not allow for a 4 week waiting period. Hospital admission at the time of MDT consultation that continued after MDT consultation was also a reason to exclude patients. Finally, patients were excluded in the ETT protocol if there was no access to the electronic patient record because the patient opted out of research use of their patient data. In case patients were treated more than once for newly onset colorectal cancer during the study period, only the first tumor treatment period was taken into analysis to avoid duplicates.

### Treatment Strategies

Usual care was compared with prehabilitation. Usual care consisted of anemia treatment as indicated (using intravenous iron medication or blood transfusion per protocol), a 30-min preoperative assessment with the physical therapist for breathing exercises, and a preoperative calculation of the nutritional assessment score (SNAQ)^17^ In case the SNAQ-score ≥ 3, patients were referred for a consultation with a dietician. In addition, patients were accompanied from diagnosis to the end of the treatment trajectory (including both surgery and chemotherapy) by a specialized oncology nurse who provided detailed information about the diagnosis and treatment process, as well as psychological support. On indication, a psychologist was consulted.

The multimodal prehabilitation consisted of case management of a specialized oncology nurse and anemia treatment, comparable to the usual care group. In addition, patients were strongly advised to reduce intoxications (smoking cessation and reduction of alcohol intake). During intake with the physical therapist, a personalized exercise program was made for each participant. Moreover, each patient received a tailored nutritional advice from a dietician.

The exercise program designed by the physical therapist contained two components: (1) three times a week, for at least 3 weeks, a 60 min high-intensity training in the hospital supervised by a physical therapist, (2) four times a week for at least 60 min, a nonsupervised low-intensity endurance training at home (e.g., walking or biking). During the intake with a dietician, patients received tailored nutritional advice to achieve a total protein intake of 1.9 g per kg of lean body mass per day. Patients were also advised to take an additional 0.4 g per kg protein, within 1 h before high-intensity training and daily before bedtime. If necessary, protein shakes were prescribed to achieve this intake. During prehabilitation, patients were followed by a dietician with a final consultation at the end of the program.

As of 2019, all patients were screened on frailty, using the G8 questionnaire.^18^ In case of a G8 score ≤ 14, patients were referred to a geriatrician for advice on delirium prevention and medication review.

Postoperative management, based on the enhanced recovery after surgery (ERAS) protocols, was the same for all patients both in the usual care and prehabilitation group.^19^

### Treatment Assignment

Ideally, in an RCT design, patients should have been blindly randomized to the usual care or prehabilitation group. However, due to the observational character of the data for this study, randomization was impossible. For the ETT, treatment assignment was based on the MDT consultation date. Eligible patients were assigned to the usual care group if MDT consultation took place before 1 June 2017 and to the prehabilitation group if MDT consultation took place after 31 October 2018 (excluding the COVID period). Next, we calculated the propensity of treatment assignment to the prehabilitation or control condition based on the predictors of treatment assignment that we could identify, and weighted our analysis for this propensity.

### Time Zero and Follow-up

Time zero was defined in the ETT protocol as the time of MDT consultation, at which a decision was made to schedule elective colorectal cancer surgery. As in the target trial protocol, the follow-up lasted up to 30 days after surgery in the ETT protocol, and in case no surgery was performed after inclusion, patients were followed for 2 months after time zero.

### Outcomes

The primary outcome was the number of patients with at least one complication (defined as yes/no complication) during follow-up. A distinction was made between complications that occurred during the total follow-up period as the primary outcome, and during the preoperative and postoperative periods as secondary outcomes. A preoperative complication was defined as any deviation from the normal preoperative course, not related to preexisting comorbidity, including but not limited to colorectal cancer (CRC)-related complications (e.g., ileus or new-onset anemia after inclusion requiring blood transfusion), prehabilitation-related complications (e.g., loss of consciousness during physical exercise or acute renal failure as a result of protein suppletion), and adverse events such as traffic accident injuries. A postoperative complication was defined as any deviation from the normal postoperative course, classified following the standards of the European Society of Anaesthesiology (ESA) and the European Society of Intensive Care Medicine (ESICM).^20^

To take into account both the number and severity of postoperative complications, the comprehensive complication score (CCS) was calculated as the secondary outcome.^21^ Finally, the length of stay (in days) and number of readmissions was compared between the standard care and the prehabilitation groups.

### Causal Contrasts

To compare the two treatment strategies, both an intention-to-treat effect as well as the per-protocol effect was estimated in the target trial. The intention-to-treat effect in the ETT included all patients eligible for prehabilitation, as well as patients who did not start the program because of another reason not described in the exclusion criteria (e.g., not motivated to participate, prehabilitation not in hospital setting, or holiday before surgery). The per-protocol effect for both the target and the ETT consisted of all patients who had at least nine consultations with the physical therapist and two consultations with the dietician.

### Statistical Analysis

Unweighted baseline characteristics of the patients in the prehabilitation versus the standard care group were compared using the student’s *t* or Mann–Whitney *U* tests for numerical variables and chi-square or Fisher’s exact tests for categorical variables, depending on data distribution.

After exploring missing data patterns and mechanisms, missing data for potential confounders were assumed to be missing at random. Missing data was mainly from the lack of electronic patient files in the beginning of 2016. To deal with missing data, baseline values of all potential confounders were imputed using a multiple imputation approach based on fully conditional specification (MICE package, R).^22^

The primary analysis followed an intention-to-treat format as specified under causal contrasts. Overlap weighting (OW), based on propensity scores (PS), was performed to reduce the impact of treatment selection bias and potential confounding factors.^23^ To compare the balance of baseline covariates between the standard care and prehabilitation groups, standardized mean differences were computed for the baseline covariates both before and after applying OW.^24,25^ To further characterize the effect of compliance with prehabilitation, a per-protocol analysis was performed.

All reported *p*-values are two-sided, and a *p*-value of < 0.05 was considered statistically significant. Statistical analysis was conducted using R4.1. Supplement 2 contains further information on the statistical analysis.

## Results

### Participants

**Fig. 1:**
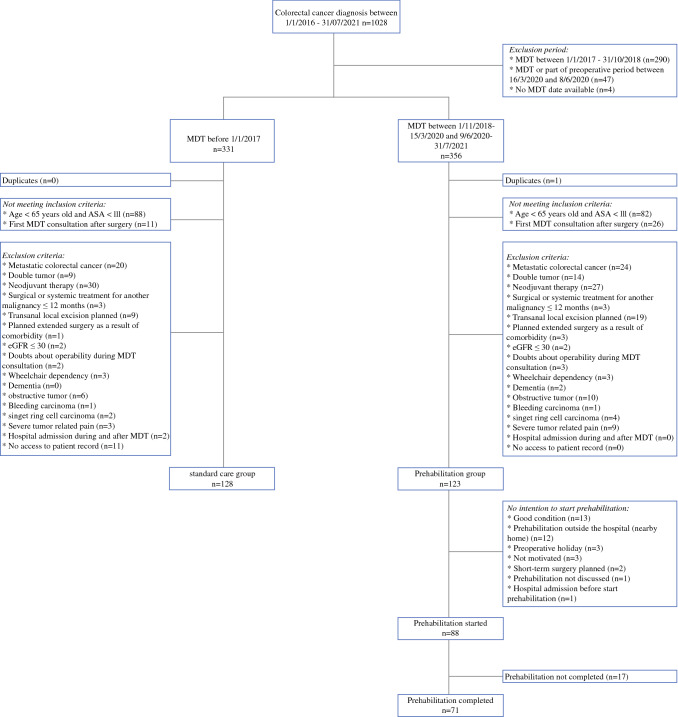
Flowchart

Between January 2016 and July 2021, 1028 patients were diagnosed with colorectal cancer (Fig. 1). After applying both inclusion and exclusion criteria, 251 patients were included in the intention-to-treat analysis (128 in the standard care group and 123 in the prehabilitation group). Baseline characteristics of the prehabilitation and control groups are presented in Table 1. Patients in the prehabilitation group were older than patients in the control group (*p* = 0.003) and less likely to use alcohol (*p* < 0.001), and had a greater incidence of anemia at the time of inclusion (*p* = 0.012). The median duration of time between inclusion and surgery was 23 [interquartile range (IQR), 17–30] days in the standard care group and 38 (IQR, 30–48) days in the prehabilitation group.

**Table 1:** Baseline characteristics of the intention-to-treat population, stratified by usual care group and prehabilitation

	Usual care (*n* = 128)	Prehabilitation (*n* = 123)	Total (*n* = 251)	*p*-Value
*Patient characteristics*				
Median age, years (IQR)	72 (68–76)	75 (71–80)	73 (69–79)	0.003
*Gender*				0.052
Male	75 (58.6%)	57 (46.3%)	132 (52.6%)	
*Civil status*				0.259
Partnership	94 (73.4%)	92 (74.8%)	186 (74.1%)	
Single	18 (14.1%)	23 (18.7%)	41 (16.3%)	
Widowed	15 (11.7%)	8 (6.5%)	23 (9.2%)	
Missing	1 (0.8%)	0 (0%)	1 (0.4%)	
*BMI, kg/m*^2^				0.319
< 18.5	2 (1.6%)	0 (0%)	2 (0.8%)	
18.5–25	48 (37.5%)	45 (36.6%)	93 (37.1%)	
25–30	51 (39.8%)	43 (35.0%)	94 (37.5%)	
≥ 30	27 (21.1%)	35 (28.5%)	62 (24.7%)	
*Smoking*				0.496
Never	58 (45.3%)	50 (40.7%)	108 (43.0%)	
Former	58 (45.3%)	65 (52.8%)	123 (49.0%)	
Current	11 (8.6%)	8 (6.5%)	19 (7.6%)	
Missing	1 (0.8%)	0 (0%)	1 (0.4%)	
*Alcohol use*				< 0.001
Never	49 (38.3%)	60 (48.8%)	109 (43.4%)	
< 1/week	28 (21.9%)	8 (6.5%)	36 (14.3%)	
≥ 1/week < 2/daily	26 (20.3%)	42 (34.1%)	68 (27.1%)	
≥ 2/daily	23 (18.0%)	13 (10.6%)	36 (14.3%)	
Missing	2 (1.6%)	0 (0%)	2 (0.8%)	
*Charlson comorbidity index*				0.621
0	66 (51.6%)	58 (47.2%)	124 (49.4%)	
1	26 (20.3%)	32 (26.0%)	58 (23.1%)	
2	24 (18.8%)	19 (15.4%)	43 (17.1%)	
≥ 3	12 (9.4%)	14 (11.4%)	26 (10.4%)	
*Polypharmacy (≥ 5 drugs)*				0.154
Yes	51 (39.8%)	60 (48.8%)	111 (44.2%)	
*ASA index*				0.171
I	10 (7.8%)	6 (4.9%)	16 (6.4%)	
II	74 (57.8%)	61 (49.6%)	135 (53.8%)	
III	40 (31.3%)	54 (43.9%)	94 (37.5%)	
IV	4 (3.1%)	2 (1.6%)	6 (2.4%)	
*MET score*				0.438
< 3	2 (1.6%)	3 (2.4%)	5 (2.0%)	
3–6	43 (33.6%)	72 (58.5%)	115 (45.8%)	
>3	40 (31.3%)	47 (38.2%)	87 (34.7%)	
Missing	43 (33.6%)	1 (0.8%)	44 (17.5%)	
*SNAQ score*				0.950
≥ 3	15 (11.7%)	16 (13.0%)	31 (12.4%)	
Missing	27 (21.1%)	13 (10.6%)	40 (15.9%)	
*Anemia at inclusion*				0.012
Yes	48 (37.5%)	66 (53.7%)	114 (45.4%)	
Missing	1 (0.8%)	0 (0%)	1 (0.4%)	
*Tumor characteristics*				
Tumor localisation				0.056
Colon	61 (47.7%)	76 (61.8%)	137 (54.6%)	
Sigmoid	48 (37.5%)	37 (30.1%)	85 (33.9%)	
Rectum	19 (14.8%)	10 (8.1%)	29 (11.6%)	
*Tumor stage*				0.114
I	36 (28.1%)	33 (26.8%)	69 (27.5%)	
II	57 (44.5%)	42 (34.1%)	99 (39.4%)	
III	35 (27.3%)	48 (39.0%)	84 (33.1%)	
*Stoma at inclusion*				0.165
Yes	2 (1.6%)	6 (4.9%)	8 (3.2%)	

*ASA* American Society of Anesthesiologists, *MET score* Metabolic Equivalent of Task score, *SNAQ score* Short Nutritional Assessment Questionnaire score

Only one patient, in the prehabilitation group, underwent open surgery; all other patients underwent laparoscopic colectomy. Conversion during surgery was needed in five patients (4%) in the standard care group and four (3%) in the prehabilitation group. In both the usual care group and the prehabilitation group, most patients underwent right hemicolectomy [53 (41%) and 60 (49%) patients, respectively]. Stoma creation was performed in 23 patients (18%) in the usual care group and 7 patients (6%) in the prehabilitation group (Table 2).

**Table 2:** Operative characteristics of the intention-to-treat population, stratified by usual care group and prehabilitation

	Usual care (*n* = 128)	Prehabilitation (*n* = 123)	Total (*n* = 251)
*Surgical approach*			
Open	0 (0%)	1 (0.8%)	1 (0.4%)
Laparoscopic	128 (100%)	122 (99.2%)	250 (99.6%)
*Conversion*			
Yes	5 (3.9%)	4 (3.3%)	9 (3.6%)
*Type of surgery*			
Right hemicolectomy	53 (41.4%)	60 (48.8%)	113 (45.0%)
Left hemicolectomy	5 (3.9%)	16 (13.0%)	21 (8.4%)
Transverse colectomy	3 (2.3%)	1 (0.8%)	4 (1.6%)
Subtotal colectomy	1 (0.8%)	1 (0.8%)	2 (0.8%)
Anterior/sigmoid resection	47 (36.7%)	35 (28.5%)	82 (32.7%)
Low anterior resection	16 (12.5%)	7 (5.7%)	23 (9.2%)
Abdominoperineal resection	3 (2.3%)	3 (2.4%)	6 (2.4%)
*Stoma creation*			
Yes	23 (18.0%)	7 (6.0%)	30 (12.0%)

There were 35 patients in the prehabilitation group who did not start in-hospital prehabilitation, because of various reasons including subjectively rating their condition as good (*n* = 13), prehabilitation program outside the hospital (*n* = 12), planned preoperative holiday (*n* = 3), no motivation to start prehabilitation (*n* = 3), short-term planned surgery (*n* = 2), prehabilitation not discussed (*n* = 1), and hospital admission before starting prehabilitation (*n* = 1) (Fig. 1). Of the patients who started in-hospital prehabilitation, 71 completed the program with at least nine in-hospital supervised exercise sessions and two consultations with a dietician (per-protocol group, baseline and operative characteristics specified in Supplement 3). Reasons not to complete prehabilitation (*n* = 17) were mainly because surgery was planned on short-term (*n* = 10). In all these cases, early surgery was the result of the hospital’s planning for the operating theater and not due to tumor-related complications. Other reasons were subjectively rated good condition (*n* = 2), prehabilitation too burdensome (*n* = 2), personal circumstances making intensive prehabilitation program unfeasible (*n* = 2), and holiday in between (*n* = 1).

No data were missing for the trial outcomes (detailed description of unweighted trial outcomes are presented in Supplement 4). Multiple imputation was applied for missing baseline characteristics, as specified in Table 1.

### Propensity Scores and Overlap Weighting

There was high overlap of PS between the usual care group and the prehabilitation group, in both the intention-to-treat analysis and the per-protocol analysis (Supplement 5).

After overlap weighting, the balance of the baseline characteristics was improved between the standard care group and prehabilitation group, both on patient characteristics (e.g., age, ASA index, and MET score) and tumor characteristics (e.g., tumor localization and tumor stage) (Supplement 6).

### Intention-to-Treat Analyses

There was a significant risk reduction in the occurrence of overall complications for patients in the prehabilitation group (weighted complication risk 0.45) compared with patients in the usual care group [weighted complication risk 0.69, absolute risk difference (ARD) −0.24 (95% CI −0.38 to −0.10), Table 3] and a significant risk reduction for postoperative complications [ARD −0.28 (95% CI −0.42 to −0.15)]. The number needed to treat (NNT) to reduce at least one or more overall and postoperative complication in one patient was 4.2 (95% CI 2.6–10) and 3.6 (95% CI 2.4–6.7), respectively. There was no significant difference in the occurrence of preoperative complications between the two groups [ARD 0.05 (95% CI −0.03 to 0.12), in favor of the prehabilitation group].

**Table 3:** Primary weighted outcomes of the intention-to-treat population, stratified by usual care group and prehabilitation

Weighted outcomes	Usual care (*n* = 128)	Prehabilitation (*n* = 123)	Weighted ARD	95% CI interval
*Complication risk*				
Total	0.69	0.45	−0.24	−0.38 to −0.10
Preoperative	0.04	0.09	0.05	−0.03 to 0.12
Postoperative	0.67	0.39	−0.28	−0.42 to −0.15

*ARD* absolute risk difference

Compared with patients in the usual care group, patients in the prehabilitation group had a 55% lower CCS score (95% CI −71% to −32%). The geometric mean CCS score based on overlap weighting was 3.2 in the prehabilitation group compared with 7.2 in the usual care group.

Compared with patients in the usual care group, patients in the prehabilitation group had a 33% lower length of stay [incidence rate ratio −0.33 (95% CI −0.44 to −0.18)]. The expected length of stay based on overlap weighting was 4.9 days in the prehabilitation group compared with 7.3 days in the standard care group.

There was no significant risk difference in the number of readmissions between the two groups [ARD −0.03 (95%CI −0.08 to 0.03)].

### Per-Protocol Analyses

The per-protocol analysis also showed a significant risk reduction of overall complications for patients who completed prehabilitation compared with patients in the usual care group [ARD −0.21 (95% CI −0.38 to −0.04)] and a significant risk reduction for postoperative complications [ARD −0.27 (95% CI −0.44 to −0.11)]. Therefore, the NNT to prevent one extra patient from having at least one overall and one postoperative complication was 4.8 (95% CI 2.6–25) and 3.7 (95% CI 2.3–9.1), respectively. There was no significant difference in the occurrence of preoperative complications between the two groups [ARD 0.06 (95% CI −0.03 to 0.16)]. Weighted complication risks are presented in Table 4.

**Table 4:** Primary weighted outcomes of the per-protocol population, stratified by usual care group and prehabilitation

Weighted outcomes	Usual care (*n* = 128)	Prehabilitation (*n* = 71)	Weighted ARD	95% CI interval
*Complication risk*				
Total	0.73	0.52	−0.21	−0.38 to −0.04
Preoperative	0.05	0.11	0.06	−0.03 to 0.16
Postoperative	0.70	0.43	−0.27	−0.44 to −0.11

Compared with patients in the usual care group, patients who had completed prehabilitation had a 55% lower CCS score (95% CI −73 to 24%). Geometric mean CCS score based on overlap weighting was 3.6 in the group who completed prehabilitation compared with 8.0 in the usual care group.

Compared with patients in the usual care group, patients who completed prehabilitation had a 22% shorter length of stay; however, this was not statistically significant [incidence rate ratio −0.22 (95% CI −0.39 to −0.01)]. The expected length of stay based on overlap weighting was 5.9 days in the group who completed prehabilitation compared with 7.6 days in the standard care group.

There was no significant absolute risk difference in the number of readmissions between the two groups [ARD −0.03 (95%CI −0.08 to 0.02)].

## Discussion

This is the first ETT assessing the impact of a prehabilitation intervention on perioperative complications in patients undergoing colorectal cancer surgery. Under the assumption that the causal model for treatment assignment was valid, the study showed that prehabilitation led to a significant risk reduction of perioperative complications. No significant difference in preoperative complications, e.g., as a result of a longer waiting time before surgery, was seen. There was a significant reduction in the number of patients who had a postoperative complication and a significant reduction in CCI scores. Furthermore, there was a significant reduction of LOS. Prehabilitation had no effect on the already limited number of readmissions.

Additional to previous RCTs that have shown evidence for the effectiveness of prehabilitation, this study provides the first preliminary evidence that prehabilitation not only improves outcomes in a controlled study setting, but also in daily clinical practice.^6,10,11^ Compared with previous studies showing no effect of prehabilitation, this study investigated a multimodal prehabilitation program including supervised high-intensity training.^12,26–28^ The concept behind combining multiple interventions, in addition to an Enhanced Recovery After Surgery (ERAS) program, is that of marginal gains.^29–31^ Although optimizing an individual aspect of care only results in a marginal gain, the aggregation effect of these marginal gains can be considerable.

The main strength of this study, compared with previous observational studies,^32–34^ is that the design and analyses are based on a target randomized controlled trial. Biases that often arise in observational studies, including confounding-by-indication and immortal time bias, were minimized through rigorous implementation of carefully selecting the participants based on the eligibility criteria, properly identifying the time zero, and performing both intention-to-treat and per-protocol effects. The minimization of immortal time bias is also of added value compared with previous RCTs in which patients who did not receive an operation were excluded from analysis after randomisation.^11,35^ In addition to patients who were potentially unfit for surgery, this study only excluded patients from the analysis that were simply not able to perform prehabilitation, such as those who were not mobile enough to participate in prehabilitation programs and those with cognitive deterioration impeding adherence to the program. Classical observational studies do not exclude or include patients with this level of rigor. This implies that observational comparisons often include patients that will never be scheduled for prehabilitation in the first place. As a result, the generalizability and transportability is increased in this study, because the study sample is more representative of patients in daily clinical practice. Moreover, the design of this study was much less resource-intensive compared with RCTs and, consequently, much more feasible in daily clinical practice. Additionally, instead of inverse probability weighting (IPW), which is often hampered by extreme propensity scores, this study used the overlap weighting (OW) method.^23^ Compared with IPW combined with propensity score trimming, OW produces an unbiased treatment effect estimate with lower variance and good coverage.^23^

There are also some limitations of the current study: (1) This is a single-center study limiting external validity. (2) The lack of random assignment may have resulted in confounded effect estimates. Although weighting was applied for all relevant baseline confounders of treatment assignment that could be measured, the findings are only internally valid if the assumed causal model for treatment assignment was correct. (3) Because a historical cohort was used for comparison, it is possible that the reduction in perioperative complications, LOS, and postoperative readmissions within 30 days after surgery was the result of better usual care over the years, rather than the effect of prehabilitation alone. However, given the relative short study period (5 years) and the previously described improvements in postoperative outcomes in the Netherlands between 2005 and 2016, it is not very likely that better usual care is the only reason for the demonstrated improvement in postoperative outcomes.^4^ (4) Measurement errors may have occurred because data were collected retrospectively from electronic patient records. It cannot be ruled out that identifying complications may vary somewhat over time. (5) Not including patients during or after neoadjuvant treatment may be a limitation. We excluded patients with neoadjuvant treatment in this study to reduce heterogeneity. Patients who have had neoadjuvant treatment have a different tumor pathology (locally advanced) and a different preoperative trajectory, with a longer preoperative waiting time and resilience disruption due to neoadjuvant treatment. (6) Because of the limited number of patients with ASA IV, it was not possible to draw any conclusion about the ability of a patient with ASA IV to perform high-intensity training repeatedly at least three times a week.

The methodology used in this study could be an example for further multicenter studies. However, to be able to use (large) clinical registration databases in future studies on the effect of prehabilitation and other complex interventions, these databases should incorporate more clinical predictor variables (e.g., frailty score), further specification of clinical outcomes (e.g., CCS score), and patient-reported outcome measures (e.g., quality of life).

In conclusion, this study showed a clinically relevant reduction of complications and LOS after a multimodal prehabilitation program in patients with a higher postoperative complication risk undergoing colorectal cancer surgery, compared with standard care. The study methodology may serve as an example for further larger multicenter comparative effectiveness research on prehabilitation.

## Supplementary Information

Below is the link to the electronic supplementary material.

Supplementary file1 (DOCX 19 kb)

Supplementary file2 (DOCX 20 kb)

Supplementary file3 (DOCX 24 kb)

Supplementary file4 (DOCX 15 kb)

Supplementary file5 (DOCX 53 kb)

Supplementary file6 (DOCX 17 kb)
