# Supplementary material for: Improved Postoperative Outcomes after Prehabilitation for Colorectal Cancer Surgery in Older Patients: An Emulated Target Trial
Source: Ann Surg Oncol. 2022 Oct 5;30(1):244–54. doi: 10.1245/s10434-022-12623-9 (PMC9533971; doi:10.1245/s10434-022-12623-9)
Supplement: Supplementary file 1 — Supplementary file1 (DOCX 19 kb) [file 10434_2022_12623_MOESM1_ESM.docx]

## Supplement 1

**Table 1 Specification and emulation of the target trial**

|  | **Target trial protocol** | **Target trial emulation protocol** |
| --- | --- | --- |
| **Eligibility criteria** |  |  |
| *Inclusion criteria* |  |  |
|  | Candidate to elective colorectal cancer surgery | Multidisciplinary team (MDT) consultation before colorectal cancer surgery, discussing the need for surgery. If the first MDT consultation was after surgery or there was no MDT consultation at all, patients were excluded. |
|  | Higher risk for surgical complications defined by: age ≥ 65 years or American Society of Anesthesiologists score III/IV | Same |
| *Exclusion criteria* |  |  |
|  | Metastatic colorectal cancer | Same |
|  | Double tumor | Same |
|  | Neoadjuvant therapy | Same |
|  | Surgical or systemic (chemo- or radiotherapy) treatment for another malignancy in the previous 12 months before MDT consultation | Same |
|  | Transanal local excision | Same |
|  | Planned extended surgery as result of comorbidity | Same |
|  | Kidney failure defined as an eGFR ≤ 30 | Same |
|  | Unstable cardiac or respiratory disease, making patient unfit for surgery | Doubts about operability (based on physical condition) during MDT consultation, making patient unfit for surgery. |
|  | Locomotor limitations precluding exercise | Wheelchair dependency |
|  | Cognitive deterioration impeding  adherence to the program | Dementia registered as comorbidity |
|  | Preoperative schedule not allowing for at least 4 weeks for the prehabilitation intervention | Reasons to plan surgery within 4 weeks after MDT consultation:   - Obstructive tumor - Bleeding carcinoma - Signet ring cell carcinoma - Severe tumor related pain   Hospital admission at the time of MDT consultation that continued after MDT consultation |
|  | No written informed consent | No access to patient record, because patient opted out of research use of their patient data |
| **Treatment strategies** | Usual care group: usual preoperative care  Prehabilitation group: usual preoperative care + prehabilitation | Same |
| **Treatment assignment** | Patients were blindly randomized to the usual care or prehabilitation group using a 1:1 ratio | Based on the multidisciplinary team (MDT) consultation date patients were assigned to the usual care (MDT consultation before 01/06/2017) or prehabilitation group (MDT consultation after 31/10/2018). |
| **Time zero and follow-up** | Time zero was defined as the time of informed consent and baseline assessment.  Individuals were followed up until 30 days after tumor resection surgery.  In case no surgery was performed after inclusion, patients were followed up during 2 months after time zero. | Time zero was defined as the time of MDT consultation  Individuals were followed up to 30 days after tumor resection surgery.  In case no surgery was performed after inclusion, patients were followed for 2 months after time zero. |
| **Outcomes** |  |  |
| *Primary* | Number of patients with a complication (including adverse events). Distinguishing total follow-up period, preoperative period and postoperative period. | Same |
| *Secondary* | Comprehensive complication score (CCS) per patient[20].  Length of stay (days).  Number of readmissions. | Same |
| **Causal contrasts** | Intention-to-treat effect  Per-protocol effect | Same |
| **Statistical analysis** | Intention-to-treat analysis.  Per-protocol analysis. | Intention-to-treat analysis including all eligible patients in both usual care as well as prehabilitation group (regardless whether patients started prehabilitation or not).  Per protocol analysis including all eligible patients in usual care group and patients who followed the prehabilitation program for at least 3 weeks.  Application overlap weighting, based on propensity scores, to adjust for baseline confounders[22]. |
