# Supplementary material for: Improved Postoperative Outcomes after Prehabilitation for Colorectal Cancer Surgery in Older Patients: An Emulated Target Trial
Source: Ann Surg Oncol. 2022 Oct 5;30(1):244–54. doi: 10.1245/s10434-022-12623-9 (PMC9533971; doi:10.1245/s10434-022-12623-9)
Supplement: Supplementary file 2 — Supplementary file2 (DOCX 20 kb) [file 10434_2022_12623_MOESM2_ESM.docx]

## Supplement 2

Unweighted baseline characteristics of the patients in the prehabilitation versus the standard care group were compared using the Student’ or Mann-Whitney U test for numerical variables and chi squire or Fisher exact tests for categorical variables, depending on data distribution.

Missing data for potential confounders were assumed to be missing at random. Missing data was mainly caused by the lack of an electronic patient file in the beginning of 2016. To deal with missing data, baseline values of all potential confounders were imputed using a multivariate imputation model (mice package R)[20]. An imputation model was used including baseline and outcome variables associated with at least one of the missing variables (imputed datasets = 20, iterations=100). Convergence plots were generated to check if the imputed values had the expected variation between the iterations.

The primary analysis followed an intention-to-treat format as specified under causal contrasts. Overlap weighting (OW), based on propensity scores (PS) was performed to reduce the impact of treatment selection bias and potential confounding factors[21]. The propensity score was estimated as the predicted probability of a patient being in the prehabilitation group from a logistic regression model, considering pretreatment variables that were prognostic for postoperative outcomes[24]. The propensity score model included age, gender, civil status, BMI, smoking status, alcohol use, Charlson Comorbidity Index (CCI), polypharmacy, American Society of Anesthesiologists index (ASA index), Metabolic Equivalent of Task score (MET-score), Short Nutritional Assessment Questionnaire score (SNAQ-score), yes or no anemia at inclusion, tumor localization, tumor stage and yes or no stoma at inclusion. Weights for patients in the prehabilitation group were the 1-PS and weights for patients in the standard care group were the PS. To compare the balance of baseline covariates between the standard care group and prehabilitation group, standardized mean differences were computed for the baseline covariates both before and after applying OW [22, 23].

Weighted logistic regression modeling was applied to analyse primary outcomes in the 20 imputation sets. A linear model with a negative binomial distribution and a logarithmic link function was used for the analysis of length-of-stay. For the comprehensive complication scores (CCS), a linear regression with the log-transformed CSS was used to account for the skewed distribution of CCS data. To account for within-subject homogeneity induced by weighting, a robust variance estimator was included in the regression models [25, 26]. The results per imputation set were combined using Rubin’s rule[27].

To further characterize the effect of compliance with prehabilitation, a per-protocol analysis was performed. For this per-protocol analysis new propensity scores and weights were calculated (based on data of all patients who had at least nine consultations with the physical therapist and two consultations with the dietician).

All reported p-values in this study are 2 sided, and a p-value of < .05 was considered statistically significant. Statistical analysis was conducted using RStudio version 1.1.463.
