# Supplementary material for: Improved Postoperative Outcomes after Prehabilitation for Colorectal Cancer Surgery in Older Patients: An Emulated Target Trial
Source: Ann Surg Oncol. 2022 Oct 5;30(1):244–54. doi: 10.1245/s10434-022-12623-9 (PMC9533971; doi:10.1245/s10434-022-12623-9)
Supplement: Supplementary file 3 — Supplementary file3 (DOCX 24 kb) [file 10434_2022_12623_MOESM3_ESM.docx]

## Supplement 3

**Table 1 Baseline characteristics of the per-protocol population, stratified by usual care group and prehabilitation group**

|  | **Usual care group (n=128)** | **Prehabilitation (n=71)** | **Total (n=199)** | **P-value** |
| --- | --- | --- | --- | --- |
| ***Patient characteristics*** |  |  |  |  |
| Median age (IQR) | 72 (68-76) | 76 (71-82) | 73 (69-79) | <0,001 |
| Gender |  |  |  | 0,489 |
| Male | 75 (58,6%) | 38 (53,5%) | 113 (56,8%) |  |
| Civil status |  |  |  | 0,205 |
| Partnership | 94 (73,4%) | 57 (80,3%) | 151 (75,9%) |  |
| Single | 18 (14,1%) | 11 (15,5%) | 29 (14,6%) |  |
| Widowed | 15 (11,7%) | 3 (4,2%) | 18 (9,0%) |  |
| Missing | 1 (0,8%) | 0 (0%) | 1 (0,5%) |  |
| BMI, kg/m2 |  |  |  | 0,487 |
| <18.5 | 2 (1,6%) | 0 (0%) | 2 (1,0%) |  |
| 18.5-25 | 48 (37,5%) | 28 (39,4%) | 76 (38,2%) |  |
| 25-30 | 51 (39,8%) | 23 (32,4%) | 74 (37,2%) |  |
| ≥30 | 27 (21,1%) | 20 (28,2%) | 47 (23,6%) |  |
| Smoking |  |  |  | 0,076 |
| Never | 58 (45,3%) | 24 (33,8%) | 82 (41,2%) |  |
| Former | 58 (45,3%) | 44 (62,0%) | 102 (51,3%) |  |
| Current | 11 (8,6%) | 3 (4,2%) | 14 (7,0%) |  |
| Missing | 1 (0,8%) | 0 (0%) | 1 (0,5%) |  |
| Alcohol use |  |  |  | 0,014 |
| Never | 49 (38,3%) | 37 (52,1%) | 86 (43,2%) |  |
| <1x/week | 28 (21,9%) | 6 (8,5%) | 34 (17,1%) |  |
| ≥1x/week - <2x/daily | 26 (20,3%) | 21 (29,6%) | 47 (23,6%) |  |
| ≥2x/daily | 23 (18,0%) | 7 (9,9%) | 30 (15,1%) |  |
| Missing | 2 (1,6%) | 0 (0%) | 2 (1,0%) |  |
| Charlson comorbidity index |  |  |  | 0,799 |
| 0 | 66 (51,6%) | 32 (45,1%) | 98 (49,2%) |  |
| 1 | 26 (20,3%) | 15 (21,1%) | 41 (20,6^) |  |
| 2 | 24 (18,8%) | 15 (21,1%) | 39 (19,6%) |  |
| ≥3 | 12 (9,4%) | 9 (12,7%) | 21 (10,%) |  |
| Polypharmacy (≥5 drugs) |  |  |  | 0,041 |
| Yes | 51 (39,8%) | 39 (54,9%) | 90 (45,2%) |  |
| ASA index |  |  |  | 0,041 |
| I | 10 (7,8%) | 2 (2,8%) | 12 (6,0%) |  |
| II | 74 (57,8%) | 32 (45,1%) | 106 (53,3%) |  |
| III | 40 (31,3%) | 36 (50,7%) | 76 (38,2%) |  |
| IV | 4 (3,1%) | 1 (1,4%) | 5 (2,5%) |  |
| MET-score |  |  |  | 0,218 |
| <3 | 2 (1,6%) | 1 (1,4%) | 3 (1,5%) |  |
| 3-6 | 43 (33,6%) | 45 (63,4%) | 88 (44,2%) |  |
| >3 | 40 (31,3%) | 24 (33,8%) | 64 (32,2%) |  |
| Missing | 43 (33,6%) | 1 (1,4%) | 44 (22,1%) |  |
| SNAQ-score |  |  |  | 0,591 |
| ≥3 | 15 (11,7%) | 8 (11,3%) | 23 (11,6%) |  |
| Missing | 27 (21,1%) | 4 (5,6%) | 31 (15,6%) |  |
| Anemia at inclusion |  |  |  | 0,006 |
| Yes | 48 (37,5%) | 41 (57,7%) | 89 (44,7%) |  |
| Missing | 1 (0,8%) | 0 (0%) | 1 (0,5%) |  |
| ***Tumor characteristics*** |  |  |  |  |
| Tumor localisation |  |  |  | 0,049 |
| Colon | 61 (47,7%) | 45 (63,4%) | 106 (53,3%) |  |
| Sigmoid | 48 (37,5%) | 22 (31,0%) | 70 (35,2%) |  |
| Rectum | 19 (14,8%) | 4 (5,6%) | 23 (11,6%) |  |
| Tumor stage |  |  |  | 0,622 |
| I | 36 (28,1%) | 19 (26,8%) | 55 (27,6%) |  |
| II | 57 (44,5%) | 28 (39,4%) | 85 (42,7%) |  |
| III | 35 (27,3%) | 24 (33,8%) | 59 (29,6%) |  |
| Stoma at inclusion |  |  |  | 0,189 |
| Yes | 2 (1,6%) | 4 (5,6%) | 6 (3,0%) |  |

Abbreviations: ASA= American Society of Anesthesiologists, MET-score= Metabolic Equivalent of Task score, SNAQ-score= Short Nutritional Assessment Questionnaire score

**Table 2 Operative characteristics of the per-protocol population, stratified by usual care group and prehabilitation group**

|  | **Standard care group (n=128)** | **Prehabilitation (n=71)** | **Total (n=199)** |
| --- | --- | --- | --- |
| Surgical approach |  |  |  |
| Open | 0 (0%) | 1 (1,4%) | 1 (0,5%) |
| Laparoscopic | 128 (100%) | 70 (98,6%) | 198 (99,5%) |
| Conversion |  |  |  |
| Yes | 5 (3,9%) | 2 (2,8%) | 7 (3,5%) |
| Type of surgery |  |  |  |
| Right hemicolectomy | 53 (41,4%) | 34 (47,9%) | 87 (43,7%) |
| Left hemicolectomy | 5 (3,9%) | 12 (16,9%) | 17 (8,5%) |
| Transverse colectomy | 3 (2,3%) | 0 (0%) | 3 (1,5%) |
| Subtotal colectomy | 1 (0,8%) | 1 (1,4%) | 2 (1,0%) |
| Anterior/ sigmoid resection | 47 (36,7%) | 20 (28,8%) | 67 (33,7%) |
| Low anterior resection | 16 (12,5%) | 2 (2,8%) | 18 (9,0%) |
| Abdominoperineal resection | 3 (2,3%) | 2 (2,8%) | 5 (2,5%) |
| Stoma creation |  |  |  |
| Yes | 23 (18,0%) | 4 (5,6%) | 27 (13,6%) |
