# Supplementary material for: Improved Postoperative Outcomes after Prehabilitation for Colorectal Cancer Surgery in Older Patients: An Emulated Target Trial
Source: Ann Surg Oncol. 2022 Oct 5;30(1):244–54. doi: 10.1245/s10434-022-12623-9 (PMC9533971; doi:10.1245/s10434-022-12623-9)
Supplement: Supplementary file 4 — Supplementary file4 (DOCX 15 kb) [file 10434_2022_12623_MOESM4_ESM.docx]

## Supplement 4

**Table 1 Detailed description of unweighted trial outcomes, stratified by usual care group and prehabilitation group**

| **Outcomes** | **Standard care group (n=128)** | **Prehabilitation (n=123)** | **Total (n=251)** |
| --- | --- | --- | --- |
| Complication; yes | 87 (68,0%) | 64 (52,0%) | 151 (60,2%) |
| *Preoperative* | *6 (4,7%)* | *23 (18,7%)* | *29 (11,6%)* |
| *Postoperative* | *85 (66,4%)* | *51 (41,5%)* | *136 (54,2%)* |
| Postoperative complication score (median, min-max) | 1 (0-16) | 0 (0-10) | 1 (0-16) |
| *Surgical complication score(median, min-max)* | *0 (0-10)* | *0 (0-8)* | *0 (0-10)* |
| *General complication score(median, min-max)* | *1 (0-8)* | *0 (0-7)* | *0 (0-8)* |
|  |  |  |  |
| Length of stay (median, IQR) | 6 (4-8) | 4 (3-5) | 5 (4-7) |
|  |  |  |  |
| Postoperative hemorrhage | 8 (6,2%) | 7 (5,7%) | 15 (6,0%) |
| Anastomotic leakage | 5 (3,9%) | 4 (3,2%) | 9 (3,6%) |
| Ileus | 13 (10,2%) | 13 (10,6%) | 26 (10,4%) |
| Abscess | 8 (6,2%) | 4 (3,2%) | 12 (4,8%) |
| Fascia dehiscence | 2 (1,6%) | 1 (0,8%) | 3 (1,2%) |
| Surgical site infection | 8 (6,2%) | 5 (4,1%) | 13 (5,2%) |
| Bowel perforation | 0 | 0 | 0 |
| Urethra leakage | 0 | 0 | 0 |
| Other surgical complications | 3 (2,3%) | 2 (1,6%) | 5 (2,0%) |
| Cardiovascular | 8 (6,2%) | 11 (8,9%) | 19 (7,6%) |
| Respiratory | 10 (7,8%) | 3 (2,4%) | 13 (5,2%) |
| Neurological | 8 (6,2%) | 4 (3,2%) | 12 (4,8%) |
| Thromboembolic | 0 | 2 (1,6%) | 2 (0,8%) |
| Infection other than respiratory or surgical | 10 (7,8%) | 2 (1,6%) | 12 (4,8%) |
| Other general complications | 67 (52,3%) | 29 (23,6%) | 90 (35,9%) |

Abbreviations: IQR= Interquartile range
