# Supplementary material for: Improved Postoperative Outcomes after Prehabilitation for Colorectal Cancer Surgery in Older Patients: An Emulated Target Trial
Source: Ann Surg Oncol. 2022 Oct 5;30(1):244–54. doi: 10.1245/s10434-022-12623-9 (PMC9533971; doi:10.1245/s10434-022-12623-9)
Supplement: Supplementary file 5 — Supplementary file5 (DOCX 53 kb) [file 10434_2022_12623_MOESM5_ESM.docx]

## Supplement 5

**Figure 1: Distribution of propensity scores for the intention-to-treat analysis**


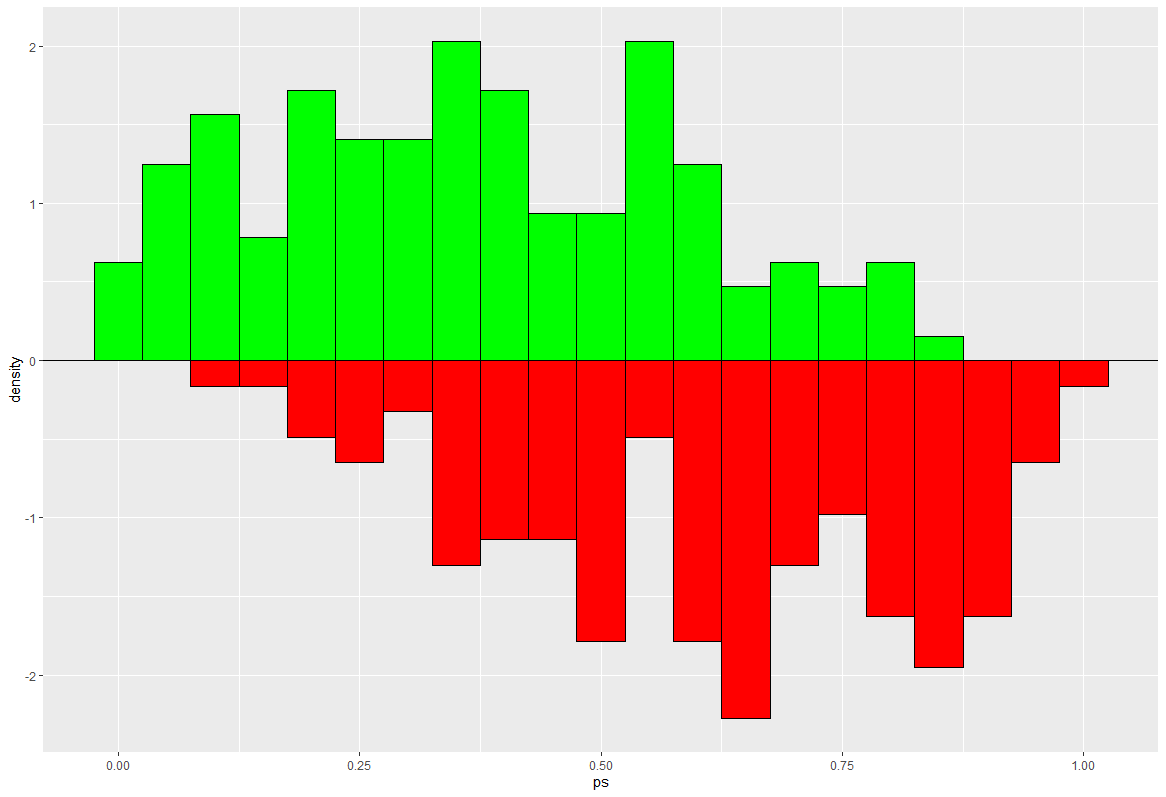


Green= standard care group, red= prehabilitation group

**Figure 2: Distribution of propensity scores for the per-protocol analysis**


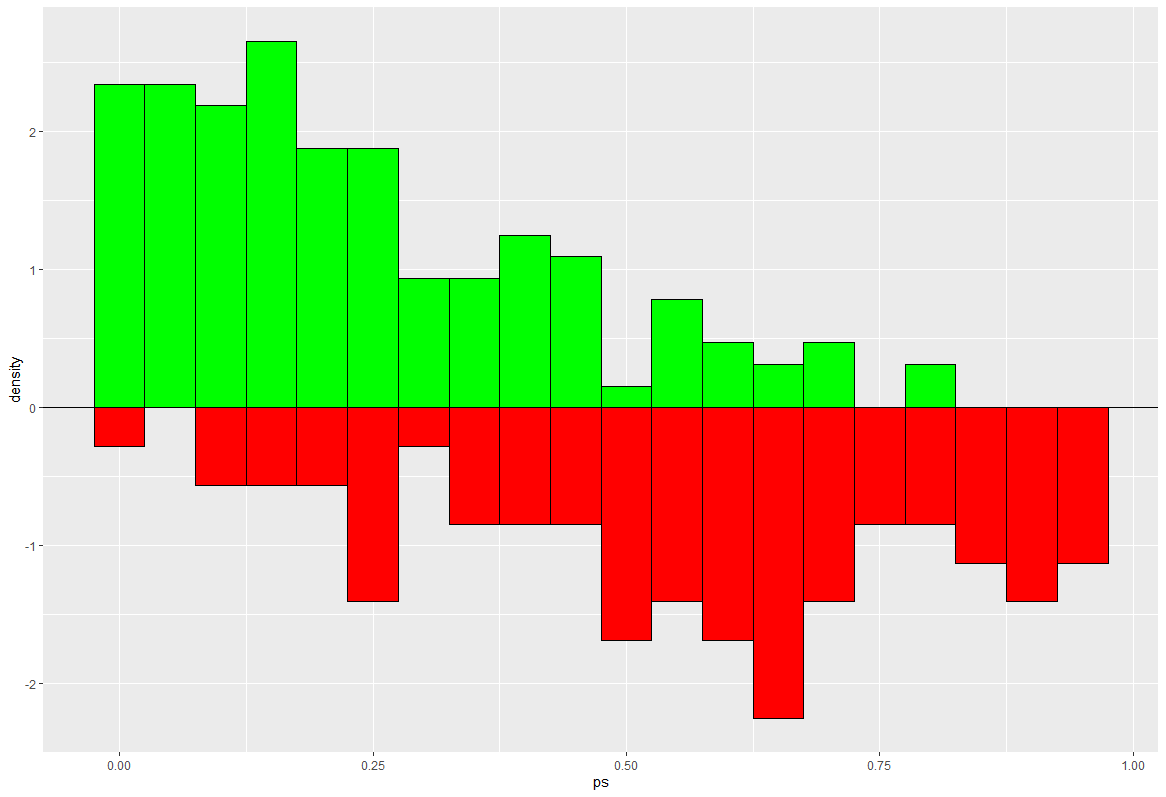


Green= standard care group, red= prehabilitation group
