# Supplementary material for: Improved Postoperative Outcomes after Prehabilitation for Colorectal Cancer Surgery in Older Patients: An Emulated Target Trial
Source: Ann Surg Oncol. 2022 Oct 5;30(1):244–54. doi: 10.1245/s10434-022-12623-9 (PMC9533971; doi:10.1245/s10434-022-12623-9)
Supplement: Supplementary file 6 — Supplementary file6 (DOCX 17 kb) [file 10434_2022_12623_MOESM6_ESM.docx]

## Supplement 6

**Table 1 Standardized mean differences of baseline covariates between standard care group versus prehabilitation group before and after overlap weighting for the intention-to-treat-analysis**

|  | **Before OW** | **After OW** |
| --- | --- | --- |
| ***Patient characteristics*** |  |  |
| Age | 0,365 | <0,001 |
| Gender | 0,246 | <0,001 |
| Civil status | 0,095 | 0,008 |
| BMI, kg/m2 | 0,187 | <0,001 |
| Smoking | 0,046 | 0,001 |
| Alcohol use | 0,104 | 0,004 |
| Charlson comorbidity index | 0,056 | <0,001 |
| Polypharmacy (≥5 drugs) | 0,180 | <0,001 |
| ASA index | 0,198 | <0,001 |
| MET-score | 0,250 | 0,051 |
| SNAQ-score | 0,018 | 0,009 |
| Anemia at inclusion | 0,327 | <0,001 |
| ***Tumor characteristics*** |  |  |
| Tumor localisation | 0,305 | <0,001 |
| Tumor stage | 0,167 | <0,001 |
| Stoma at inclusion | 0,188 | <0,001 |

Abbreviations: ASA= American Society of Anesthesiologists, MET-score= Metabolic Equivalent of Task score, SNAQ-score= Short Nutritional Assessment Questionnaire score, OW= overlap weighting

**Table 2 Standardized mean differences of baseline covariates between standard care group versus prehabilitation group before and after overlap weighting for the per-protocol-analysis**

|  | **Before OW** | **After OW** |
| --- | --- | --- |
| ***Patient characteristics*** |  |  |
| Age | 0,530 | <0,001 |
| Gender | 0,102 | <0,001 |
| Civil status | 0,227 | 0,007 |
| BMI, kg/m2 | 0,178 | <0,001 |
| Smoking | 0,125 | 0,002 |
| Alcohol use | 0,187 | 0,007 |
| Charlson comorbidity index | 0,111 | <0,001 |
| Polypharmacy (≥5 drugs) | 0,304 | <0,001 |
| ASA index | 0,339 | <0,001 |
| MET-score | 0,328 | 0,022 |
| SNAQ-score | 0,028 | 0,054 |
| Anemia at inclusion | 0,412 | <0,001 |
| ***Tumor characteristics*** |  |  |
| Tumor localisation | 0,375 | <0,001 |
| Tumor stage | 0,102 | <0,001 |
| Stoma at inclusion | 0,219 | <0,001 |

Abbreviations: ASA= American Society of Anesthesiologists, MET-score= Metabolic Equivalent of Task score, SNAQ-score= Short Nutritional Assessment Questionnaire score, OW= overlap weighting
